# A novel estimator for the two-way partial AUC

**DOI:** 10.1186/s12911-023-02382-2

**Published:** 2024-02-20

**Authors:** Elias Chaibub Neto, Vijay Yadav, Solveig K. Sieberts, Larsson Omberg

**Affiliations:** Sage Bionetworks, 2901 Third Avenue, 98121 Seattle, USA

**Keywords:** ROC curve, AUC, Partial AUC, Diagnostic testing, Machine learning performance metric

## Abstract

**Background:**

The two-way partial AUC has been recently proposed as a way to directly quantify partial area under the ROC curve with simultaneous restrictions on the sensitivity and specificity ranges of diagnostic tests or classifiers. The metric, as originally implemented in the tpAUC R package, is estimated using a nonparametric estimator based on a trimmed Mann-Whitney U-statistic, which becomes computationally expensive in large sample sizes. (Its computational complexity is of order $O(n_x n_y)$, where $n_x$ and $n_y$ represent the number of positive and negative cases, respectively). This is problematic since the statistical methodology for comparing estimates generated from alternative diagnostic tests/classifiers relies on bootstrapping resampling and requires repeated computations of the estimator on a large number of bootstrap samples.

**Methods:**

By leveraging the graphical and probabilistic representations of the AUC, partial AUCs, and two-way partial AUC, we derive a novel estimator for the two-way partial AUC, which can be directly computed from the output of any software able to compute AUC and partial AUCs. We implemented our estimator using the computationally efficient pROC R package, which leverages a nonparametric approach using the trapezoidal rule for the computation of AUC and partial AUC scores. (Its computational complexity is of order $O(n \log n)$, where $n = n_x + n_y$.). We compare the empirical bias and computation time of the proposed estimator against the original estimator provided in the tpAUC package in a series of simulation studies and on two real datasets.

**Results:**

Our estimator tended to be less biased than the original estimator based on the trimmed Mann-Whitney U-statistic across all experiments (and showed considerably less bias in the experiments based on small sample sizes). But, most importantly, because the computational complexity of the proposed estimator is of order $O(n \log n)$, rather than $O(n_x n_y)$, it is much faster to compute when sample sizes are large.

**Conclusions:**

The proposed estimator provides an improvement for the computation of two-way partial AUC, and allows the comparison of diagnostic tests/machine learning classifiers in large datasets where repeated computations of the original estimator on bootstrap samples become too expensive to compute.

## Background

### Motivation

Diagnostic tests based on continuous scales play a critical role in clinical practice, where at-risk individuals from a population are often screened for a disease using the results of quantitative laboratory tests [1]. Similarly, computer aided diagnostic technologies based on machine learning [2] (ML) are increasingly becoming an useful tool to aid clinicians in their decision making process [3–7].

However, before a new diagnostic test or ML based diagnostic classifier can be deployed in clinical practice it is essential to evaluate its accuracy and compare its performance against established methods. For diagnostic tests based on continuous measurements, a positive test result is obtained when the measurement is above a given threshold. Similarly, for ML based classifiers, a positive diagnosis is obtained when the ML algorithm prediction (usually reported as the probability that the individual has the disease) is above a given threshold. The receiver operation characteristic curve (ROC) [8] plots the false positive rate (FPR) against the the true positive rate (TPR) across all possible threshold levels and is a widely used technique for visualizing the trade-offs between FPR and TPR at any threshold level of interest in both diagnostic testing based on continuous scales [9–13] and machine learning evaluations [8]. The area under the ROC curve (AUC) is the most widely used criterium to summarize the information provided by the ROC curve across all thresholds.

For every diagnostic test/classifier there is a cost-benefit analysis necessary for evaluating the costs of false positives versus false negatives. These costs can sometimes be measured economically as in unnecessary medical care costs for false positives, but also translate to societal and individual costs such as psychological strain due to false positive diagnosis or the ethical concerns due to the direct harm caused by a false negative diagnosis (which might lead to poor prognosis or even the death of the individual). As a consequence, in many applications, it is important to simultaneously maintain a low FPR and high TPR, and regulatory or policy making bodies may have pre-defined criteria for the allowable TPR and FPR in various scenarios based on the healthcare economics of false positive and false negative outcomes. For example, the World Health Organization (WHO) has set minimal requirements for community-based tuberculosis screening at sensitivity (TPR) above 90% and specificity (1-FPR) above 70% [14]. For SARS-CoV-2 diagnostics, the WHO requirements for antigen-detecting rapid diagnostic tests are sensitivity above 80% and specificity above 97% [15]. Thus, in evaluating diagnostic tests/classifiers, we may only be interested in comparing their performances within the regulatory guideline bounds.

When clear guidelines exist, there is an advantage to compare different classifiers in a limited region of the ROC space instead of over the entire range.

### The current two-way partial AUC estimator, and it’s limitations

The two-way partial AUC [16] (tpAUC) has been proposed as a way to directly quantify the area under the ROC curve satisfying explicit constraints in both the TPR and FPR ranges. Previous approaches in the literature [17–21] focused on quantifying partial area under the ROC (pAUC) by directly restricting the FPR range, but only indirectly controlling for the TPR. However, as illustrated in [16], this indirect approach can be problematic making diagnostic test comparisons less efficient and, in some circumstances, leading to incorrect conclusions (see the illustrative examples presented in [16] for further details).

Yang et al. [16] proposed a non-parametric estimator for the tpAUC based on a trimmed version of the Mann-Whitney U statistic [22] and implemented the method in the R [23] package tpAUC. Based on this estimator, the authors also described a bootstrap [24] based testing approach, based on a asymptotic confidence interval, to compare two tpAUCs. In practice, an important caveat of the tpAUC estimator proposed by [16] (which is this paper will be denoted as the “original estimator”, $\widehat{tpAUC}_o$) is that it is computationally expensive to calculate for large datasets. (Its computational complexity is of order $O(n_x \, n_y)$, where $n_x$ and $n_y$ represent the number of positive and negative cases in the dataset, respectively.) Furthermore, because the statistical approach for comparing two tpAUCs requires bootstrap resampling, the approach can quickly become computationally impractical as sample sizes increase.

### Our contribution

To circumvent this problem, in this paper we propose a computationally efficient estimator of tpAUC, denoted as the “proposed estimator”, $\widehat{tpAUC}_p$, which can be directly computed from the output of any software able to compute the standard (“full”) AUC and the standard partial AUC metrics [17], which only impose restrictions in either the FPR (specificity) or the TPR (sensitivity) ranges, but not on both simultaneously. We implemented our estimator using the computationally efficient pROC [25] R package, which implements a nonparametric approach based on trapezoids, rather than (trimmed) Mann-Whitney U statistic estimators, for the computation of AUC and partial AUC scores. We compare the computation time and the empirical bias of the original and proposed estimators in a series of simulation studies. Furthermore, we use two real datasets to illustrate the computational efficiency of the proposed estimator (as opposed to the original one) when performing the statistical comparison of two tpAUCs from different classifiers built on the same dataset (which requires bootstrapping).

Our empirical comparisons show that the proposed estimator tended to be less biased than the original one across all experiments (and showed considerably less bias in the experiments based on small sample sizes). But, most importantly, it can be orders of magnitude faster to compute than the original estimator when sample sizes are large.

### Technical background

Before we present our proposed estimator, in the rest of this section we first review the mathematical definitions and key properties of the concepts of: (i) area under the curve (AUC); (ii) partial area under the curve (pAUC); and (iii) two-way partial area under the curve (tpAUC). We start by introducing some notation.

#### Notation

Throughout the text *X* represents a continuous test result for the positive cases, e.g., subjects that have a disease, while *Y* represents the test result for the negative cases, e.g., non-disease subjects. (In the context of machine learning applications, *X* and *Y* represent the confidence score, i.e., the predicted probability of the positive class, generated by a ML algorithm.) For any threshold *c*, a subject is classified as a positive case if its test result (confidence score) is larger than *c*. Let *F*(*x*) and *G*(*y*) represent, respectively, the cumulative distribution functions of the *X* and *Y* variables, while $S_F(x)$ and $S_G(y)$ represent the respective survival functions. Then the true positive rate (TPR) at a threshold *c*, denoted by *TPR*(*c*), is defined as,

1 $$\begin{aligned} TPR(c) \equiv P(X > c) \equiv S_F(c) = 1 - F(c)~, \end{aligned}$$

while the corresponding false positive rate (FPR) at the threshold *c*, denoted by *FPR*(*c*), is defined as,

2 $$\begin{aligned} FPR(c) \equiv P(Y > c) \equiv S_G(c) = 1 - G(c)~. \end{aligned}$$

Note the TPR is also denoted as sensitivity (SENS) of the test/classifier, while the specificity (SPEC) corresponds to $1 - \textrm{FPR}$, so that $SENS(c) = TPR(c)$ and $SPEC(c) = 1- FPR(c)$.

Throughout the text we let $D_k = \{X_i, Y_j\}$, $i = 1, \ldots , n_x$, $j = 1, \ldots , n_y$, represent a dataset containing $n_x$ positive cases and $n_y$ negative cases, and we assume that the cases are statistically independent of each other. We let $\hat{S}_{F,n_x}$ and $\hat{S}_{G,n_y}$ represent, respectively, the empirical versions of the survival functions $S_{F}$ and $S_{G}$, so that for any $u \in [0, 1]$ we have that $\hat{S}^{-1}_{F,n_x}(u) = X_{(\lfloor (1-u)n_x \rfloor )}$ and $\hat{S}^{-1}_{G,n_y}(u) = Y_{(\lfloor (1-u)n_y \rfloor )}$, where $X_{(i)}$ and $Y_{(j)}$ represent the associated order statistics, and $\lfloor N \rfloor$ represents the largest integer smaller than *N*.

#### The area under the ROC curve (AUC)

**Fig. 1:**
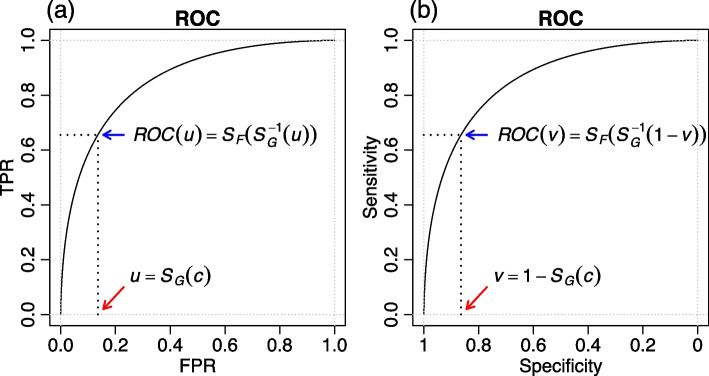
ROC curves. Panels **a** and **b** show, respectively, the ROC curve defined in terms of FPR/TPR and specificity/sensitivity. In this illustrative example, the ROC curve was parameterized according to the binormal ROC model [18], where $X \sim N(1.5, 1)$, $Y \sim N(0, 1)$, $S_F(c) = \Phi ((\mu _x - c)/\sigma _x) = \Phi (1.5 - c)$, $S_G(c) = \Phi ((\mu _y - c)/\sigma _y) = \Phi (-c)$, and $\Phi (.)$ represents the cumulative distribution function of a standard normal random variable. (Further details about the binormal ROC model are presented in the Bias comparison section.) In this example, we adopt $c = 1.1$, so that the FPR value in panel a (red arrow) is $u = S_G(1.1) = 0.136$ and the respective ROC value (blue arrow) is $ROC(0.136) = \Phi \left(1.5 - S_G^{-1}(0.136)\right) = 0.655$

For any given threshold *c*, the costs and benefits of a diagnostic test (or classifier) are associated with a given FPR/TPR (specificity/sensitivity) pair. The ROC curve plots the $\{FPR(c), TPR(c)\}$ pairs for all possible threshold values *c* (as illustrated in Fig. 1a) and provides a visual description of the trade-offs between sensitivity and specificity as *c* changes. (Equivalently, the ROC curve can also be defined in terms of specificity and sensitivity by plotting $\{SPEC(c), SENS(c)\}$ for all possible threshold values *c*, as shown in Fig. 1b.)

Mathematically, for any given threshold *c* we can describe the ROC curve as a function of the respective FPR value $u = S_G(c)$ as $ROC(u) = S_F(c) = S_F\left(S_G^{-1}(u)\right)$, where $S_G^{-1}(.)$ represents the inverse function of the survival function $S_G(.)$. Figure 1a provides an illustrative example where the red arrow represents the FPR value (*u*) associated with the threshold *c* while the blue arrow shows the respective ROC value as a function of *u*. (Note that *c* represents a threshold with values ranging in the support of the *X* and *Y* variables and, contrary to *u*, it can assume values outside the [0, 1] range.)

Often times, it is unclear how to choose a threshold *c* and it is desirable to adopt a summary measure that aggregates information across all possible threshold values. The AUC, defined as,

3 $$\begin{aligned} AUC = \int _0^1 ROC(u) \, du = \int _0^1 S_F\left(S_G^{-1}(u)\right) \, du~, \end{aligned}$$

summarizes information across all thresholds and is the most commonly used metric of accuracy in diagnostic tests (and is also widely adopted in machine learning applications). A well known statistical property of the AUC is that it corresponds to the probability that a classifier will rank a randomly chosen positive case higher than a randomly chosen negative one, that is $AUC = P(X > Y)$ [8, 11].

Empirical (nonparametric) AUC estimates can be efficiently computed from empirical ROC curves using the sum of trapezoids [8] as implemented in the pROC [25] R package. (As described in [8], the computational complexity of the AUC estimation is of order $O(n \log n)$, where $n = n_x + n_y$.) Importantly, it has been shown by Bamber [26] that, in the special case that the continuous test results (or confidence scores) have no ties, the AUC can also be estimated using the Mann-Whitney U-statistic [22],

4 $$\begin{aligned} \widehat{AUC} = \frac{1}{n_x \, n_y} \sum \limits _{i=1}^{n_x} \sum \limits _{j=1}^{n_y} 1\!\!1 \{ X_i > Y_j \}~, \end{aligned}$$

where $n_x$ and $n_y$ represents the number of positive and negative cases, respectively, and $1\!\!1\{ A \}$ represents an indicator function assuming value 1 if event *A* occurs and 0 otherwise. (This estimator, however, has computational complexity of order $O(n_x n_y)$.)

#### The partial area under the curve (pAUC)

In some applications, the interest does not lie in the entire range of FPR (or TPR) values, but in only a portion of the ROC curve. In these situations, we can use the partial area under the curve (pAUC) [17, 18, 27, 28] to summarize the information across the portion of the ROC curve that are of interest. As described in [17], the pAUC can be defined both in terms of restricting the FPR (or specificity) ranges or the TPR/sensitivity ranges. When focusing on the FPR range $[u_0, u_1]$ the pAUC is defined as [17],

5 $$\begin{aligned} pAUC_{fpr}(u_0, u_1){} & {} = \int _{S_G^{-1}(u_1)}^{S_G^{-1}(u_0)} P(X > z) \, g(z) \, dz \end{aligned}$$

6 $$\begin{aligned}{} & {} = P\left[X > Y, S_G^{-1}(u_1) \le Y \le S_G^{-1}(u_0)\right]~. \end{aligned}$$

Note that the partial AUC focusing on FPR can be re-expressed in terms of specificity as, $pAUC_{fpr}(u_0, u_1) = pAUC_{sp}(1 - u_0, 1 - u_1)$.

When focusing on sensitivity, the pAUC focusing on the TPR range $[u'_0, u'_1]$ is given by [17],

7 $$\begin{aligned} pAUC_{se}\left(u'_0, u'_1\right){} & {} = \int _{S_F^{-1}(u'_1)}^{S_F^{-1}(u'_0)} P(Y < z) \, f(z) \, dz \end{aligned}$$

8 $$\begin{aligned}{} & {} = P\left[X > Y, S_F^{-1}\left(u'_1\right) \le X \le S_F^{-1}\left(u'_0\right)\right]~. \end{aligned}$$

Similarly to the full AUC, empirical (nonparametric) partial AUC estimates can be efficiently computed from empirical ROC curves. In the pROC R package, partial AUCs are computed by ignoring trapezoids outside the partial range and adding partial trapezoids with linear interpolation when necessary [25]. Furthermore, as described in [17], the partial AUCs can also be estimated nonparametrically using trimmed versions of the Mann-Whitney U-statistic as,

9 $$\begin{aligned} \widehat{pAUC}_{fpr}(u_{0}, u_{1}) = \frac{1}{n_{x} \, n_{y}} \sum\limits_{i=1}^{n_{x}} \sum\limits_{j=1}^{n_{y}} 1\!\!1 \left\{ X_{i} > Y_{j},\right. \\ \hspace{3.5cm}\left.\hat{S}_{G,n_{y}}^{-1}(u_{1}) \le Y_{j} \le \hat{S}_{G,n_{y}}^{-1}(u_{0}) \right\}~, \end{aligned}$$

10 $$\begin{aligned} \widehat{pAUC}_{se}\left(u'_0, u'_1\right) = \frac{1}{n_x \, n_y} \sum\limits _{i=1}^{n_x} \sum\limits _{j=1}^{n_y} 1\!\!1 \left\{ X_i > Y_j,\right. \\ \hspace{3.5cm}\left. \hat{S}_{F,n_x}^{-1}(u'_1) \le X_i \le \hat{S}_{F,n_x}^{-1}(u'_0) \right\}~. \end{aligned}$$

#### The two-way partial area under the curve (tpAUC)

**Fig. 2:**
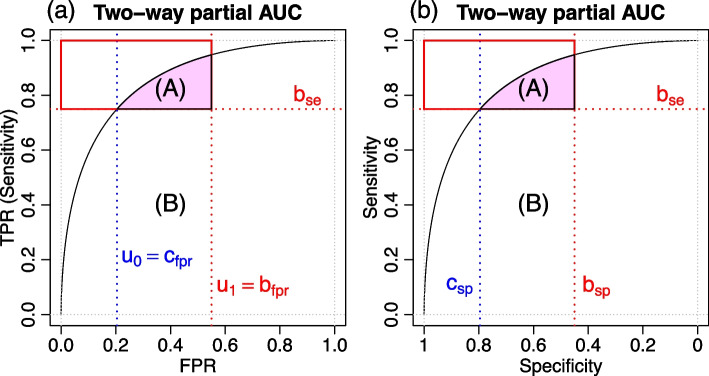
Graphical representation of the two-way partial AUC. In both panels, Area A represents the tpAUC. Panel a shows the ROC curve defined in terms of FPR and TPR (sensitivity) where the sum of Areas **A** and **B** represents the partial AUC focusing on the FPR, and $u_0 = c_{fpr} = S_G\left(S_F^{-1}(b_{se})\right)$ corresponds to the FPR value at the sensitivity threshold of $b_{se}$ and $u_1 = b_{fpr} = 1 - b_{sp}$. Panel b shows the ROC curve defined in terms of specificity and sensitivity where the sum of Areas A and B represents the partial AUC focusing on specificity, and $c_{sp} = 1 - S_G\left(S_F^{-1}(b_{se})\right)$ corresponds to the specificity value at the sensitivity threshold of $b_{se}$

In practice, we are often interested in areas of the ROC space where a diagnostic test performs above pre-specified sensitive and specificity thresholds or, equivalently, above a pre-specified TPR and below a pre-specified FPR. The tpAUC, first proposed in [16], represents an intuitive performance measure which captures partial area under the ROC with explicit restrictions on both TPR and FPR. For instance, suppose that we are only interested in sensitivity (TPR) values above the threshold $b_{se}$, and specificity values above the threshold $b_{sp}$ (or, equivalently, on FPR values below the threshold $b_{fpr} = 1 - b_{sp}$), so that we are only interested in the area in the ROC space confined to the red rectangle in Fig. 2. Then, the tpAUC as defined in [16] corresponds to the area under the ROC curve (Area A) inside the red rectangle.

From Fig. 2a, we see that, graphically, the tpAUC (Area A) corresponds to the $pAUC_{fpr}(u_0, u_1)$ (Area A + Area B) minus the Area B,

11 $$\begin{aligned} tpAUC = \underbrace{(\text {Area A + Area B})}_{pAUC_{fpr}(u_0, u_1)} - \underbrace{\text {Area B}}_{(u_1 - u_0) b_{se}} \end{aligned}$$

where $u_1 = 1 - b_{sp} = b_{fpr}$ corresponds to the upper bound on the FPR and $u_0 = c_{fpr} = S_G\left(S_F^{-1}(b_{se})\right)$ corresponds to the FPR value associated with the sensitivity bound $b_{se}$. Hence, from Eq. (5), we have that the tpAUC can be expressed mathematically as,

12 $$\begin{aligned} tpAUC(1-b_{sp}, b_{se}) {} & {} = \underbrace{\int _{S_G^{-1}(1 - b_{sp})}^{S_F^{-1}(b_{se})} S_F(z) \, g(z) \, dz}_{\text {Area A + Area B}} \; - \nonumber \\ {} & {} - \; \underbrace{[1 - b_{sp} - S_G(S_F^{-1}(b_{se}))] b_{se}}_{\text {Area B}}~, \end{aligned}$$

since $$S_G^{-1}(u_1) = S_G^{-1}\left(1 - b_{sp}\right)$$ and $$S_G^{-1}(u_0) = S_G^{-1}\left(S_G\left(S_F^{-1}(b_{se})\right)\right) = S_F^{-1}(b_{se})$$.

As described in [16], the tpAUC can also be interpreted probabilistically as,

13 $$\begin{aligned} P\left[X > Y, X \le S_F^{-1}(b_{se}) , Y \ge S_G^{-1}(1 - b_{sp})\right]~, \end{aligned}$$

and can be estimated nonparametrically using the trimmed Mann-Whitney U statistic estimator,

14 $$\begin{aligned} \widehat{tpAUC}_o = \frac{1}{n_x \, n_y} \sum \limits _{i=1}^{n_x} \sum \limits _{j=1}^{n_y} 1\!\!1 \left\{ X_i > Y_j, \right.\\ \hspace{0.5cm}\left. X_i \le \hat{S}_{F,n_x}^{-1}(b_{se}), Y_j \ge \hat{S}_{G,n_y}^{-1}(1 - b_{sp}) \right\}~, \end{aligned}$$

implemented in the tpAUC R package.

Furthermore, reference [16] also describes a bootstrap assisted approach for computing asymptotic confidence intervals for the difference between two tpAUCs (so that, different diagnostic tests/classifiers constructed in the same dataset can be compared statistically).

## Methods

In this section we describe the proposed tpAUC estimator (as well as, an alternative estimator that can be marginally faster to compute).

### The proposed tpAUC estimator ($tpAUC_p$)

**Fig. 3:**
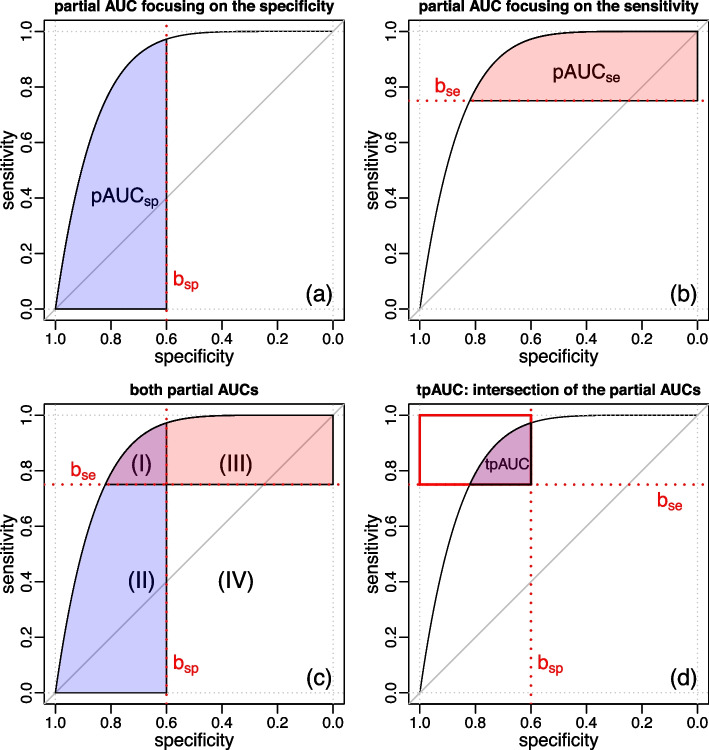
Graphical representation of the partial AUCs and the two-way partial AUC. **a** Partial AUC focusing on specificity ($pAUC_{sp}$), showing the area restricted to specificity values greater or equal to 0.6 (i.e., $b_{sp} = 0.6$). **b** Partial AUC focusing on sensitivity ($pAUC_{se}$), showing the area restricted to sensitivity values greater or equal to 0.75 (i.e., $b_{se} = 0.75$). **c** Simultaneous representation of both $pAUC_{sp}$ and $pAUC_{se}$. **d** Two-way partial AUC (*tpAUC*) as the intersection of $pAUC_{sp}$ and $pAUC_{se}$

Here, we describe a novel (and straight forward) estimator of tpAUC that can be directly computed from the output of any software that can estimate $pAUC_{sp}$, $pAUC_{se}$ and the total *AUC*. For its derivation, consider the graphical representations (and respective probabilistic interpretations) of the *AUC*, $pAUC_{sp}$, $pAUC_{se}$, and *tpAUC* quantities presented in Fig. 3.

Figure 3a shows the graphical representation of $pAUC_{sp}$ restricted to the specificity interval $[1, b_{sp}]$ (blue area), while Fig. 3b shows the graphical representation of $pAUC_{se}$ restricted to the sensitivity interval $[b_{se}, 1]$ (red area). (To simplify the exposition we drop the integration intervals from the notation and represent $pAUC_{sp}(1, b_{sp})$ by $pAUC_{sp}$ and $pAUC_{se}(b_{se}, 1)$ by $pAUC_{se}$). Figure 3c shows the representation of both quantities, simultaneously, and clearly shows that the *tpAUC* (colored area in Fig. 3d) corresponds to the intersection of the $pAUC_{sp}$ (blue area) and $pAUC_{se}$ (red area) depicted in Area I.

From Fig. 3c, we also have that,

15 $$\begin{aligned} pAUC_{sp}{} & {} = \text {Area I + Area II}~, \end{aligned}$$

16 $$\begin{aligned} pAUC_{se}{} & {} = \text {Area I + Area III}~, \end{aligned}$$

17 $$\begin{aligned} AUC{} & {} = \text {Area I + Area II + Area III + Area IV}~, \end{aligned}$$

18 $$\begin{aligned} \text {Area IV}{} & {} = b_{sp} \, b_{se}~, \end{aligned}$$

so that it is easy to see that,

19 $$\begin{aligned} tpAUC{} & {} = pAUC_{sp} + pAUC_{se} - (AUC - b_{se} \, b_{sp}) \\{} & {} = (\text {Area I + Area II}) + (\text {Area I + Area III}) - \nonumber \\{} & {} \hspace{0.5cm}- (\text {Area I + Area II + Area III + Area IV} - \text {Area IV}) \nonumber \\{} & {} = \text {Area I}~. \nonumber \end{aligned}$$

**Fig. 4:**
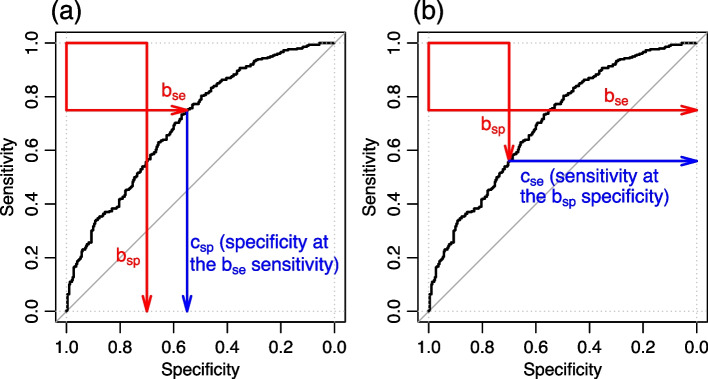
Conditions under which $tpAUC = 0$. Panel **a** shows that because the ROC curve is monotonic it follows that the curve will not cross the region of interest (red rectangle) if the specificity at the $b_{se}$ sensitivity, denoted by $c_{sp}$ and highlighted by the blue arrow, is smaller than $b_{sp}$. Panel **b** shows the equivalent condition in terms of sensitivity values, where we see that the ROC curve does not cross the region of interest if the sensitivity at the $b_{sp}$ specificity, $c_{se}$, is smaller than $b_{se}$. When these conditions hold, we have that the tpAUC is 0. In our implementation, we check the condition in panel a

Hence, we see that *tpAUC* can be computed according to Eq. (19) whenever the intersection of the $pAUC_{sp}$ and $pAUC_{se}$ areas is not empty, but will be zero whenever the $pAUC_{sp}$ and $pAUC_{se}$ areas do not intersect or, equivalently, if the ROC curve does not pass through the area of interest in the ROC space defined by the upper left corner rectangle corresponding to sensitivity values greater or equal to $b_{se}$ and specificity values greater or equal to $b_{sp}$ (e.g., the red rectangle in Fig. 3d). As described in Fig. 4a, this condition can be tested by simply checking whether the specificity value corresponding to the sensitivity threshold $b_{se}$, defined mathematically as, $c_{sp} = 1 - S_G(S_F^{-1}(b_{se}))$, is smaller than the specificity threshold $b_{sp}$. Therefore, an alternative expression for the *tpAUC* is,

20 $$\begin{aligned} tpAUC = \left\{ \begin{array}{lccl} 0 &{} , &{} \textrm{if} &{} c_{sp} < b_{sp} \\ pAUC_{se} + pAUC_{sp} - (AUC - b_{se} \, b_{sp}) &{} , &{} \textrm{if} &{} c_{sp} \ge b_{sp}~. \end{array} \right. \end{aligned}$$

Observe that Eq. (20) has the following probabilistic interpretation. Let $E_1$ and $E_2$ represent the following events,

21 $$\begin{aligned} E_1 = \left\{ X > Y, Y \ge S_G^{-1}(1 - b_{sp}) \right\}~, \end{aligned}$$

22 $$\begin{aligned} E_2 = \{ X > Y, X \le S_F^{-1}(b_{se}) \}~, \end{aligned}$$

then, if events $E_1$ and $E_2$ are not mutually exclusive, it follows from the definition of the probability of the intersection of two events that,

23 $$\begin{aligned} \underbrace{P(E_1 \cap E_2)}_{tpAUC(1-b_{sp}, b_{se})} = \underbrace{P(E_1)}_{pAUC_{sp}} + \underbrace{P(E_2)}_{pAUC_{se}} - \underbrace{P(E_1 \cup E_2)}_{AUC - b_{se} \, b_{sp}}~, \end{aligned}$$

where it is easy to see that $P(E_1 \cup E_2) = AUC - b_{se} \, b_{sp}$ since $E_1 \cup E_2$ corresponds, graphically, to the colored area (i.e., Area I + Area II + Area III) in Fig. 3c that can also be reexpressed as $\text {(Area I + Area II + Area III + Area IV) - Area IV} = AUC - b_{se} \, b_{sp}$. On the other hand, if $E_1$ and $E_2$ are mutually exclusive than $P(E_1 \cap E_2) = tpAUC = 0$.

The expression for tpAUC in Eq. (20) immediately suggests the following estimator,

24 $$\begin{aligned} \widehat{tpAUC}_p = \left\{ \begin{array}{lccl} 0 &{} , &{} \textrm{if} &{} \hat{c}_{sp} < b_{sp} \\ \widehat{pAUC}_{se} + \widehat{pAUC}_{sp} - \left(\widehat{AUC} - b_{se} \, b_{sp}\right) &{} , &{} \textrm{if} &{} \hat{c}_{sp} \ge b_{sp} \end{array} \right. \end{aligned}$$

where $\hat{c}_{sp} = 1 - \hat{S}_{G,n_y}(\hat{S}_{F,n_x}^{-1}(b_{se}))$ corresponds to the estimated specificity at the sensitivity bound $b_{se}$ (in our implementation we use the coords function from the pROC R package to compute this value).

### An alternative tpAUC estimator ($tpAUC_a$)

From Fig. 2, it is easy to see that an alternative way to compute the tpAUC (area A) using the output of any software that can estimate $pAUC_{fpr}$ and also $c_{fpr}$ (i.e., the FPR value that corresponds to the sensitivity threshold $b_{se}$) is to directly estimate the tpAUC by subtracting the area B (the rectangle given by $(b_{fpr} - c_{fpr}) b_{se}$) from the $pAUC_{fpr}(c_{fpr}, b_{fpr})$ which corresponds to the sum of areas A and B. In other words, we can directly estimate tpAUC using the alternative estimator,

25 $$\begin{aligned} \widehat{tpAUC}_a = \left\{ \begin{array}{lccl} 0 &{} , &{} \textrm{if} &{} \hat{c}_{fpr} > b_{fpr} \\ \widehat{pAUC}_{fpr}\left(\hat{c}_{fpr}, b_{fpr}\right) - \left(b_{fpr} - \hat{c}_{fpr}\right) b_{se} &{} , &{} \textrm{if} &{} \hat{c}_{fpr} \le b_{fpr} \end{array} \right. ~, \end{aligned}$$

or alternatively in terms of specificity as,

26 $$\begin{aligned} \widehat{tpAUC}_a = \left\{ \begin{array}{lccl} 0 &{} , &{} \mathrm{if} &{} \hat{c}_{sp} < b_{sp} \\ \widehat{pAUC}_{sp}\left(\hat{c}_{sp}, b_{sp}\right) - \left(\hat{c}_{sp} - b_{sp}\right) b_{se} &{} , &{} \mathrm{if} &{} \hat{c}_{sp} \ge b_{sp} \end{array} \right. ~, \end{aligned}$$

where $\hat{c}_{sp} = 1 - \hat{c}_{fpr}$. Since this alternative estimator does not require the computation of the *AUC* and $pAUC_{se}$ its computation can be potentially faster than of $tpAUC_p$.

## Results

In this section we present four sets of experiments comparing the tpAUC estimators described in this paper. We start by presenting some initial comparisons (based on simulated data) between the original and proposed estimators, followed by more systematic comparisons of the computation time requirements of the original, proposed, and alternative estimators, and a more systematic comparison of the bias of the original and proposed estimators. In our final set of experiments, we use two real datasets to illustrate the computational complexity of the original estimator when performing the statistical comparison of two tpAUCs from different classifiers built on the same dataset.

### Initial comparisons

We initially compared the original estimator based on the trimmed Mann-Whitney U statistic estimator, $\widehat{tpAUC}_o$, presented in Eq. (14) against the proposed estimator, $\widehat{tpAUC}_p$, presented in Eq. (24) in 9 initial experiments encompassing all the combinations of sample sizes set to 100, 1,000 or 10,000 cases and the proportion of negative and positive cases set to (90%, 10%), (50%, 50%), and (10%, 90%), as described in Table 1.

**Table 1:** Simulation experiments. $n_x$ and $n_y$ represent the number of positive and negative cases, respectively

	$n_y$	$n_x$	*n*	Panel in Fig. 5
experiment 1	90	10	100	(a)
experiment 2	50	50	100	(b)
experiment 3	10	90	100	(c)
experiment 4	900	100	1,000	(d)
experiment 5	500	500	1,000	(e)
experiment 6	100	900	1,000	(f)
experiment 7	9,000	1,000	10,000	(g)
experiment 8	5,000	5,000	10,000	(h)
experiment 9	1,000	9,000	10,000	(i)

**Fig. 5:**
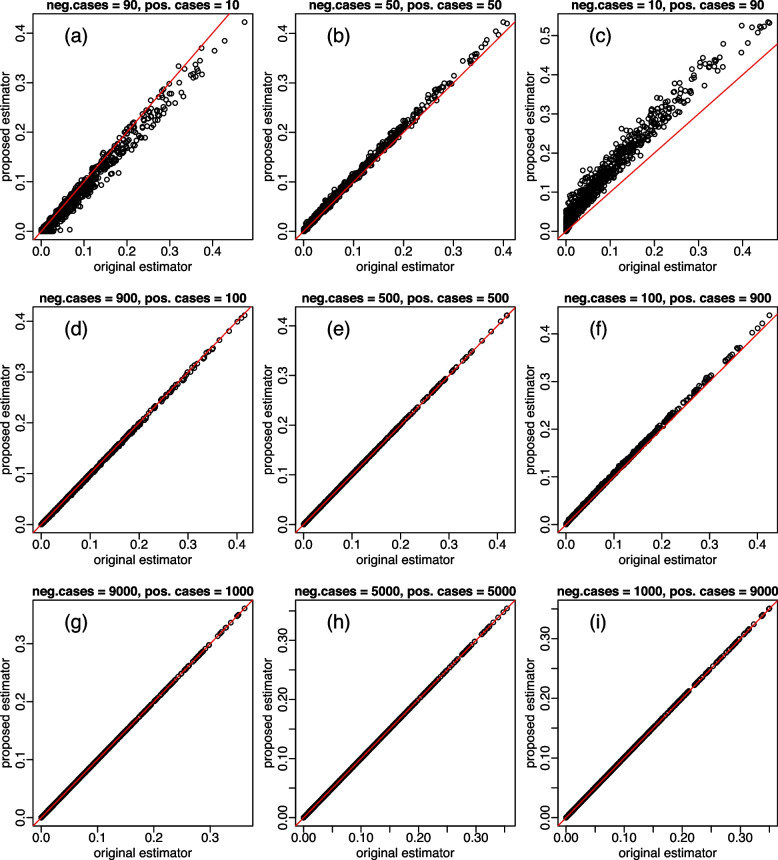
Comparison the tpAUC estimates. Panels **a** to **i** present scatterplots of the tpAUC estimates based on the original (x-axis) and proposed (y-axis) estimators across the nine initial experiments described in Table 1. The estimates tend to get very close (bottom panels) as sample size increases. Results from each experiment were based on 1,000 replications (see main text for details)

**Fig. 6:**
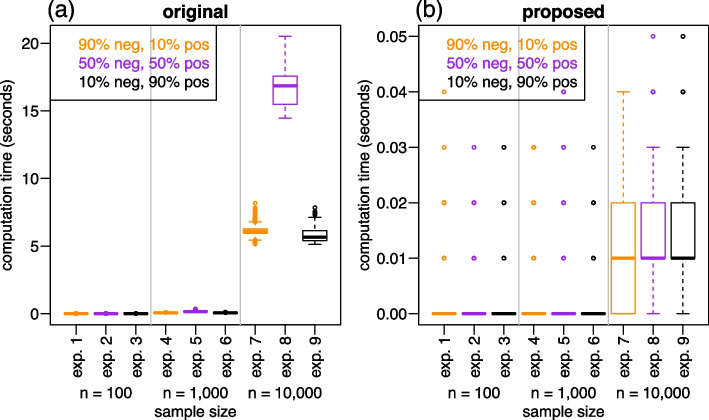
Computation time comparison. Panel **a** shows boxplots of the computation time for the original estimator for all 9 experiments grouped by their sample sizes and color coded according to the proportion of negative and positive cases. Panel **b** shows the analogous results for the proposed estimator. Note the different scales in the y-axis of panels **a** and **b**. The boxplots report the computation time across the same 1,000 replications of each one of the 9 experiments presented in Fig. 5 (and described in Table 1)

Each simulation experiment was composed of 1,000 replications where the data from each replication was generated from the binormal ROC model [18] using a different set of parameters randomly sampled from the distributions, $\mu _y \sim U(0, 2)$, $\mu _x \sim \mu _y + U(0.1, 1.1)$, $\sigma _y \sim U(0.5, 1.5)$, and $\sigma _x \sim U(0.5, 1.5)$, and the sensitivity and specificity thresholds where randomly selected from the distributions $b_{se} \sim U(0.2, 0.8)$ and $b_{sp} \sim U(0.2, 0.8)$. The tpAUC estimates and computation time associated with these initial experiments are reported in Figs. 5 and 6.

Figure 5 presents scatterplots of the tpAUC estimates obtained with the original and proposed estimators. While the estimates tend to be very close for larger sample sizes (see Fig. 5d, e, and f, and, especially, Fig. 5g, h, i), we observed a fair amount of discrepancy for the estimates based on small sample sizes (Fig. 5a, b, and c) and, in particular, for unbalanced proportions of negative and positive cases (Fig. 5a and c). These initial experiments suggest that either one or both of these estimators tended to be fairly biased for small sample sizes. Additional simulation experiments conclude that it is actually the original estimator that shows higher bias than the proposed estimator in this case (see the “Bias comparison” subsection below).

Figure 6 presents the computation times for the original (Fig. 6a) and proposed (Fig. 6b) estimators. (Computation times correspond to the “user” time reported by the system.time function from R base.) Each panel reports boxplots for all 9 experiments grouped by their sample sizes ($n = 100$ for experiments 1, 2, and 3, $n = 1,000$ for experiments 4, 5, and 6, and $n = 10,000$ for experiments 7, 8, and 9) and also color coded according to the proportion of negative and positive cases (orange boxplots for experiments with 90%/10% negative/positive cases, purple boxplots for experiments with 50%/50% negative/positive cases, and black boxplots for experiments with 10%/90% negative/positive cases). All experiments reported in this section (and throughout the paper) were performed on a Windows machine with processor Intel(R) Core(TM) i7-7820HQ CPU @ 2.90GHz 2.90 GHz and 64 GB of RAM. The computation time reported for the $\widehat{tpAUC}_p$ estimator corresponds to the sum of the time taken for: (i) estimating the ROC curve; (ii) estimating the specificity value corresponding to the sensitivity threshold (for checking whether the ROC crosses the area of interest); and (iii) the estimation of the $\widehat{pAUC}_{se}$, $\widehat{pAUC}_{sp}$, and $\widehat{AUC}$ quantities.

Figure 6a shows a very sharp increase in computation time for the original estimator when the sample size increases to 10,000. Interestingly, we observed that, within each sample size group, the original estimator tended to spent considerably more time to compute the results of the experiments based on a balanced proportion of negative and positive cases (purple boxplots) relative to the experiments with unbalanced proportions (orange and black boxplots). (E.g., for $n = 10,000$, note how the original estimator took much longer to compute the results from experiment 8 compared to experiments 7 and 9.)

On the other hand, Fig. 6b shows that the computation times for the proposed estimator show only a slight increase in computation time with increasing sample sizes, and only take a fraction of a second to compute the tpAUC even for $n = 10,000$ (note the different scales in the y-axis of Fig. 6a and b). Also, observe that the quantized computation times observed in Fig. 6b (at 0.00, 0.01, 0.02, 0.03, 0.04, and 0.05 seconds) represent an artifact of the precision with which timing results are reported by the system.time function. Despite this coarse precision, the results still clearly illustrate that the computational complexity of the proposed estimator is considerably smaller than the original one. In the next subsection, we present some additional computation time experiments.

### Additional computation time comparisons

**Fig. 7:**
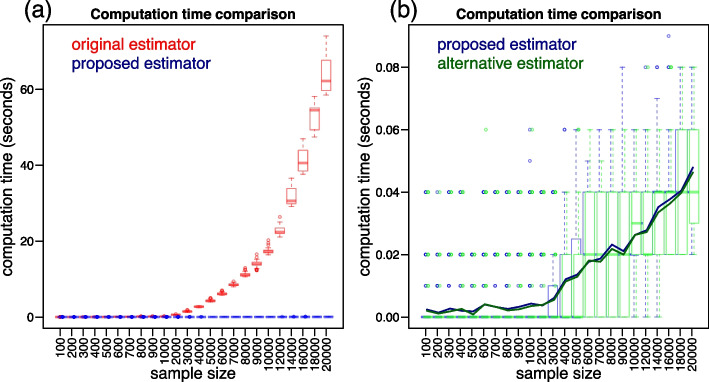
Additional computation time experiments for sample sizes increasing from 100 to 20,000. Panel **a** reports boxplots (over 100 replications) of the computation times taken by the original (red) and proposed (blue) estimators. Panel **b** presents the analogous comparison between the proposed (blue) and the alternative (green) estimators

To more systematically compare the computation times of original and proposed estimators we performed additional computation time experiments spanning a wider range of sample sizes. Additionally, we compare the proposed estimator (Eq. 24) to the alternative estimator, described in Eq. (26), which can be potentially faster to compute. (For this alternative estimator, the reported computation time corresponds to the sum of the time taken for: estimating the ROC curve; estimating the specificity value corresponding to the sensitivity threshold; and estimating of the $\widehat{pAUC}_{sp}$ if necessary. Note, as well, that we only report the computation time comparison because the estimates generated by the alternative and proposed estimators are identical.) We performed two sets of experiments. In the first, we investigated the computation times over 24 simulation experiments across a grid of sample sizes ranging from 100 to 20,000. In these first experiments, we simulated data from the binormal ROC model [18] adopting $\mu _x = 2$, $\sigma _x = 1.5$, $\mu _y = 0$, $\sigma _y = 1$, $b_{sp} = 0.6$, and $b_{se} = 0.6$, with an equal proportion of positive and negative cases and each experiment was replicated 100 times. Figure 7 reports the results. Panel a compares the original (red) and proposed (blue) estimators and shows a sharp increase in computation time for the original estimator (it can take over 1 minute for $n = 20,000$) while the proposed estimator still takes only a fraction of a second at this sample size. Panel b compares the proposed (blue) and the alternative (green) estimators. The blue and green solid lines represent the average computation time across the 100 experiment replications and shows that the alternative estimator tended to be slightly faster on average. This difference, however, is very small. (Note the small scale in the y-axis.)

**Fig. 8:**
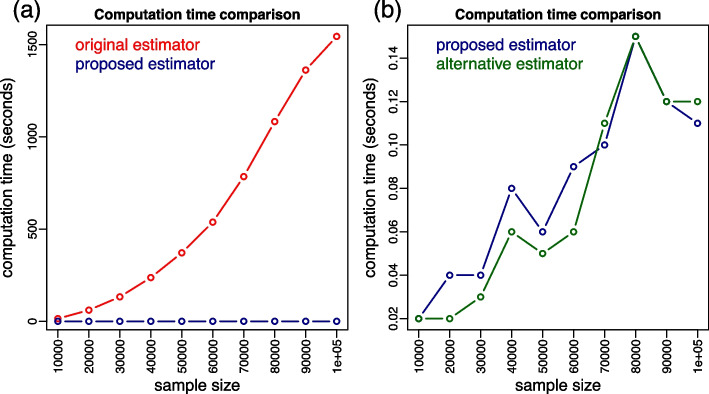
Additional computation time experiments for sample sizes increasing from 10,000 to 100,000. Panel **a** reports the computation times taken by the original (red) and proposed (blue) estimators. Panel **b** presents the analogous comparison between the proposed (blue) and the alternative (green) estimators

In the second set of experiments, we investigated the computation times over sample sizes ranging from 10,000 to 100,000. The data was generated as in the first series of experiments, except that, due to the long computation times required by the original estimator, we only performed a single replication per sample size. Figure 8 presents the results and panel a shows that the original estimator can take over 25 minutes (1,500 seconds) to compute for $n = 100,000$, while panel b shows that the computation times of the proposed and alternative estimators are comparable and take only a fraction of the time required by the original estimator (with the alternative estimator tending to be only slightly faster).

### Bias comparison

As illustrated in the initial experiments based on small sample sizes (Fig. 5 a, b, and c) the tpAUC estimates generated by the original and proposed estimators can differ substantially suggesting that one (or both) of them might be fairly biased for small sample sizes. In order to investigate this issue, we systematically compared the bias of the estimators in a series of simulation experiments over a range of sample sizes and proportions of negative and positive cases across 16 different combinations of sensitivity and specificity thresholds. But before we describe the simulation study design and results, we first describe how we estimated the bias from the tpAUC estimators.

By definition, the bias of an estimator $\hat{\theta }$ is given by,

27 $$\mathrm{Bias}(\widehat\theta)=E\lbrack\widehat\theta\rbrack-\theta\;,$$

where $\theta$ corresponds to the true parameter value. In order to compare the bias of the original and proposed tpAUC estimators, we conducted a Monte Carlo study where we simulate multiple datasets from a binormal ROC model [18] and compare the average of the empirical estimates against the true tpAUC value.

Under the binormal ROC model, the positive and negative cases are distributed, respectively, according to $X \sim N\left(\mu _x , \sigma _x^2\right)$ and $Y \sim N\left(\mu _y , \sigma _y^2\right)$ so that the true positive and false positive rates are given respectively by,

28 $$\begin{aligned} S_F(c) {} & {} \equiv P(X > c) = 1 - P(X \le c) \nonumber \\ {} & {} = 1 - \Phi \left( \frac{c - \mu _x}{\sigma _x}\right) = \Phi \left( \frac{\mu _x - c}{\sigma _x}\right) ~, \end{aligned}$$

29 $$\begin{aligned} S_G(c) {} & {} \equiv P(Y > c) = 1 - P(Y \le c) \nonumber \\ {} & {} = 1 - \Phi \left( \frac{c - \mu _y}{\sigma _y}\right) = \Phi \left( \frac{\mu _y - c}{\sigma _y}\right) ~, \end{aligned}$$

where $\Phi (.)$ represents the cumulative distribution function of a standard normal random variable.

Re-expressing the general definition of the $pAUC_{fpr}$ in Eq. (12) in terms of the binormal ROC model we have that,

30 $$\begin{aligned} {} & {} \text {(Area A + Area B)} = pAUC_{fpr}(u_0, u_1) \nonumber \\ {} & {} = \int _{S_G^{-1}(1 - b_{sp})}^{S_F^{-1}(b_{se})} S_F(z) \, g(z) \, dz \nonumber \\{} & {} = \frac{1}{\sigma _y} \int _{\mu _y - \sigma _y \Phi ^{-1}(1 - b_{sp})}^{\mu _x - \sigma _x \Phi ^{-1}(b_{se})} \Phi \left( \frac{\mu _x - z}{\sigma _x}\right) \, \phi \left( \frac{\mu _y - z}{\sigma _y}\right) \, dz~, \end{aligned}$$

where $\phi (.)$ represents the probability density function of a standard normal variable, and the integration limits in the above equation follow from the fact that, $u = S_G(z) = \Phi ((\mu _y - z)/\sigma _y)$, so that $z = S_G^{-1}(u) = \mu _y - \sigma _y \Phi ^{-1}(u)$, and $u' = S_F(z') = \Phi ((\mu _x - z')/\sigma _x)$, so that $z' = S_F^{-1}(u') = \mu _x - \sigma _x \Phi ^{-1}(u')$. Hence, $S_G^{-1}(1 - b_{sp}) = \mu _y - \sigma _y \Phi ^{-1}(1 - b_{sp})$ and $S_F^{-1}(b_{se}) = \mu _x - \sigma _x \Phi ^{-1}(b_{se})$.

Similarly, the Area B in Eq. (12) is re-expressed as,

31 $$\begin{aligned} \text {Area B}{} & {} = \left[1 - b_{sp} - S_G\left(S_F^{-1}(b_{se})\right)\right] b_{se} \nonumber \\{} & {} = \left[ 1 - b_{sp} - \Phi \left( \frac{\mu _y - (\mu _x - \sigma _x \Phi ^{-1}(b_{se}))}{\sigma _y}\right) \right] b_{se}~, \end{aligned}$$

and the true tpAUC is obtained by solving the integral in Eq. (30) using numerical integration and computing the Area B in Eq. (31) analytically, and then subtracting the latter value from the former.

We compared the bias of the original and proposed estimators in 6 simulation experiments with sample sizes set to either 100 or 1000 and with the proportion of negative and positive cases set to (90%, 10%), (50%, 50%), and (10%, 90%), as described in Table 2.

**Table 2:** Simulation experiments. $n_x$ and $n_y$ represent the number of positive and negative cases, respectively

	experiment 1	experiment 2	experiment 3	experiment 4	experiment 5	experiment 6
$n_y$	90	50	10	900	500	100
$n_x$	10	50	90	100	500	900

**Fig. 9:**
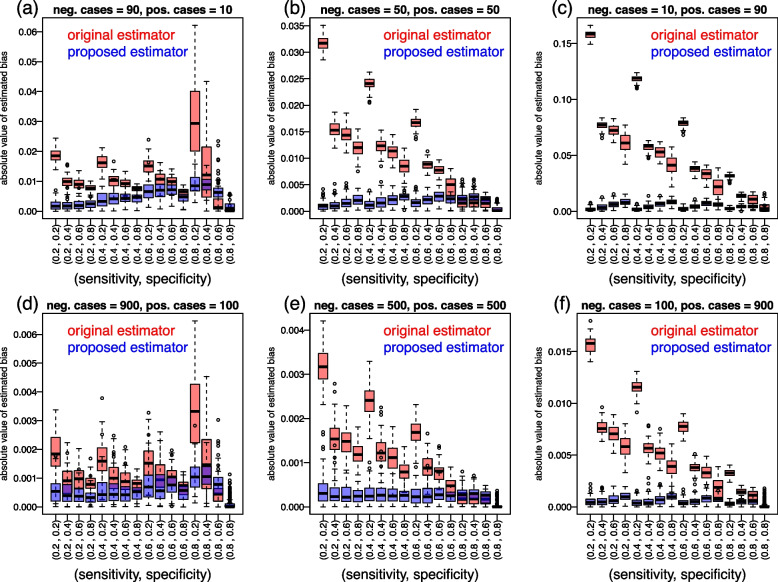
Bias comparison experiments. Each panel presents boxplots reporting the absolute value of the estimated bias for the original (red) and proposed (blue) estimators (across 100 replications of each experiment described in Table 2) for 16 different combinations of sensitivity and specificity thresholds

Each experiment was composed of 100 distinct runs where for each run we:

1. Randomly sampled the parameter values from the binormal ROC model from the following uniform distributions: $\mu _x \sim U(2, 3)$, $\sigma _x \sim U(1, 2)$, $\mu _y \sim U(0, 1)$, and $\sigma _y \sim U(1, 2)$;
2. For each of the 16 different combinations of sensitivity and specificity thresholds, $(SENS, SPEC) = \{ (0.2, 0.2)$, (0.2, 0.4), (0.2, 0.6), (0.2, 0.8), (0.4, 0.2), (0.4, 0.4), (0.4, 0.6), (0.4, 0.8), (0.6, 0.2), (0.6, 0.4), (0.6, 0.6), (0.6, 0.8), (0.8, 0.2), (0.8, 0.4), (0.8, 0.6), $(0.8, 0.8) \}$, we did the following:
  - Computed the true tpAUC value from the binormal ROC model using Eqs. (30) and (31);
  - Generated 1000 datasets $D_k = \{ X_{i}, Y_{j} \}$, $i = 1, \ldots , n_x$, $j = 1, \ldots , n_y$, from the binormal ROC model [18] where $X_i$ and $Y_j$ are randomly drawn from the normal distributions $X_i \sim N\left(\mu _x, \sigma _x^2\right)$ and $Y_j \sim N\left(\mu _y, \sigma _y^2\right)$, and for each simulated dataset $D_k$ we computed and stored the empirical tpAUC estimates $\widehat{tpAUC}_{o,k}$ (Eq. 14) and $\widehat{tpAUC}_{p,k}$ (Eq. 24);
  - Estimated the bias of the original and proposed tpAUC estimators according to,
    32 $$\begin{aligned} \widehat{\textrm{Bias}}(\widehat{tpAUC}) = \frac{1}{K} \sum \limits _{k=1}^{K} \widehat{tpAUC}_{k} - tpAUC~. \end{aligned}$$

Figure 9 reports the results from these experiments. Each panel presents boxplots (across the 100 replications) of the absolute value of the estimated bias against the 16 different combinations of sensitivity and specificity thresholds. Overall the results suggest that the original estimator (red boxplots) tends to be more biased than the proposed one (blue boxplots). Observe, as well, that the amount of bias is considerably smaller for the experiments based on larger sample size ($n = 1,000$), presented in Fig. 9d, e, and f, when compared to the experiments based on smaller samples ($n = 100$) presented in Fig. 9a, b, and c. (Note the different scale in the y-axis for the top and bottom panels in Fig. 9).

### Comparing the predictions of distinct classifiers

When comparing distinct classifiers built on the same dataset, the difference between the performance metric is a popular criterium for selecting the best classifier. To this end, Yang et al. [16] describe how to compute asymptotic confidence intervals for the difference of tpAUCs statistic,

33 $$\begin{aligned} \widehat{\Delta tpAUC} = \widehat{tpAUC}_{C_1} - \widehat{tpAUC}_{C_2}~, \end{aligned}$$

where $\widehat{tpAUC}_{C_1}$ and $\widehat{tpAUC}_{C_2}$ represent the estimates obtained by the classifiers $C_1$ and $C_2$, respectively.

However, because the predictions of classifiers trained in the same dataset tend to be correlated, the statistics $\widehat{tpAUC}_{C_1}$ and $\widehat{tpAUC}_{C_2}$ are not independent and the asymptotic theory for the statistic $\widehat{\Delta tpAUC}$ is non-standard. To circumvent this problem, Yang et al. [16] (and [17] in the context of the pAUC metric) described a bootstrap-assisted approach to calculate asymptotic confidence intervals for the $\widehat{\Delta tpAUC}$ statistic. Namely, the $100 (1 - \alpha )$% confidence interval is given by,

34 $$\begin{aligned} \left( \widehat{\Delta tpAUC} - Z_{1-\alpha /2} \, \sqrt{\frac{v^2_{boot}}{n_x + n_y}} \; , \; \widehat{\Delta tpAUC} + Z_{1-\alpha /2} \, \sqrt{\frac{v^2_{boot}}{n_x + n_y}} \right) \end{aligned}$$

where $Z_{1-\alpha /2}$ corresponds to the $1-\alpha /2$ quantile of a standard normal distribution and $v^2_{boot}$ represents the nonparametric bootstrap [24] estimate of the variance of $\widehat{\Delta tpAUC}$,

35 $$\begin{aligned} v^2_{boot} = \frac{1}{B} \sum \limits _{i = 1}^{B} \left( \widehat{\Delta tpAUC}_i - B^{-1} \sum \limits _{r=1}^{B} \widehat{\Delta tpAUC}_r \right) ^2~, \end{aligned}$$

and *B* represents the number of bootstrap samples.

Clearly, because the bootstrap estimate $v^2_{boot}$ requires the estimation of the tpAUC metric *B* times for each classifier $C_1$ and $C_2$, we have that this procedure becomes computationally impractical for larger datasets when we use the original trimmed Mann-Whitney U statistic estimator in Eq. (14).

We illustrate this point using the Diabetic Retinopathy [29] and the Sepsis Survival [30] UCI datasets [31]. The Diabetic Retinopathy is a moderate size dataset ($n = 1,151$) containing features extracted from the Messidor image set [32], including quality assessment, pre-screening, lesion-specific and anatomical components. The classification task is to predict the presence or absence of diabetic retinopathy. The Sepsis Survival is a large dataset containing 110,341 sepsis hospitalization records on 84,811 subjects. Each record contains the age, gender, and the sepsis episode number (since some individuals had multiple episodes of sepsis). To make sure that all data points were independent we only used the first record of sepsis of each individual in our analyses (so that $n = 84,811$). The classification task was to predict if a subject died or survived based on their age and gender.

**Fig. 10:**
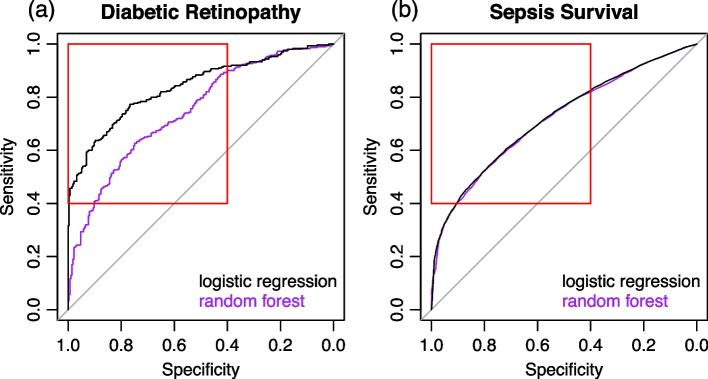
Real data illustrations. Panel **a** compares the ROC curves generated from the predictions of a logistic regression and a random forest classifier on the Diabetic Retinopathy Debrecen dataset, while panel **b** presents the analogous comparison on the Sepsis Survival dataset. In both panels the red square captures the area of interest of the ROC space obtained by setting the sensitivity and specificity thresholds to $b_{se} = 0.4$ and $b_{sp} = 0.4$, respectively

For each of these datasets we trained a logistic regression [33] and a random forest [34] classifier (using 50/50 training/test data split) and then compute 95% confidence intervals for the $\Delta tpAUC$ statistic,

36 $$\begin{aligned} \widehat{\Delta tpAUC} = \widehat{tpAUC}_{\text {logistic regression}} - \widehat{tpAUC}_{\text {random forest}}~, \end{aligned}$$

using the original and the proposed estimators of tpAUC. (We adopted the default tuning parameter values implemented in the ranger [35] R package for the random forest classification.) For both datasets we adopt sensitivity and specificity thresholds set to 0.4 and Fig. 10 presents the ROC curves based on the predicted probabilities from the logistic regression and random forest classifiers. Table 3 reports the respective confidence intervals and the time spent in their calculation (alongside the estimates of tpAUC using the logistic regression and random forest classifiers for both the original and proposed estimators). The reported results are based on $B = 1,000$ sequentially run bootstraps for the estimation of $v^2_{boot}$.

**Table 3:** Real data experiments. CI stands for confidence interval and *rf* and *lr* stand for random forest and logistic regression, respectively

UCI dataset	Diabetic Retinopathy	Sepsis Survival
total number of cases on test set	575	42,405
number of negative cases on test set	275	39,418
number of positive cases on test set	300	2,987
logistic regr. $\widehat{tpAUC}_{lr}$ (original estimator)	0.216667	0.134550
logistic regr. $\widehat{tpAUC}_{lr}$ (proposed estimator)	0.214788	0.123094
random forest $\widehat{tpAUC}_{rf}$ (original estimator)	0.138909	0.124622
random forest $\widehat{tpAUC}_{rf}$ (proposed estimator)	0.137091	0.121070
$\widehat{\Delta tpAUC}$ (original estimator)	0.077758	0.009929
$\widehat{\Delta tpAUC}$ (proposed estimator)	0.077697	0.002024
95% Confidence Interval (original estimator)	(0.076342, 0.079173)	(0.009889, 0.009968)
95% Confidence Interval (proposed estimator)	(0.076326, 0.079068)	(0.002019, 0.002029)
95% CI computation time (original estimator)	5.13 minutes	5.12 days
95% CI computation time (proposed estimator)	3.24 seconds	46.69 seconds

Comparison of the estimates based on the original and proposed estimators show that the results are fairly close, and indicate that the logistic regression classifier is statistically better than the random forest classifier, since for both datasets the 95% confidence intervals do not contain 0. (Note that for the Sepsis Survival dataset, while the difference in the tpAUC estimates is very small, the difference is still statistically significant since the very large sample size leads to a very narrow confidence interval.)

Despite the fact that the results based on the original and proposed estimators are quite similar, the computation times are drastically different. While the computation of the confidence interval based on the original estimator takes less than 6 minutes for the Diabetic Retinopathy dataset ($n = 575$), it takes over 5.12 days for the Sepsis Survival dataset ($n = 42,405$). On the other hand, the computation time based on the proposed estimator increases from 3.24 seconds in the Diabetic Retinopathy dataset to only 46.69 seconds in the Sepsis Survival dataset.

## Discussion

It is well understood that the nonparametric estimators of AUC and pAUC tend to be systematically biased in small sample sizes, especially when the ROC operating points are not well spread out along the ROC curve [36]. However, as pointed in the literature [17, 36, 37] the amount of bias becomes negligible as the sample size increases. Our bias comparison experiments (Fig. 9) also show a drastic reduction in bias when sample size increases from 100 to 1,000 for both the original and proposed estimators suggesting that this observation also holds true for the tpAUC. The fact that the proposed estimator tended to show smaller amounts of bias in comparison to the original estimator might be due to the fact that the pROC R package performs linear interpolation for adding partial trapezoids (when necessary) during the calculation of the partial AUCs.

But most importantly, our experiments and real data illustrations show that the computation of the proposed estimator can be orders of magnitude faster than the original one. This result is not surprising given that the calculation of the proposed estimator leverages the computationally efficient routines implemented in the pROC R package (see the pROC package documentation for details). Note that the computational complexity of the proposed estimator in Eq. (24) is log-linear since its computation depends on the estimation of the ROC curve and of the AUC and partial AUCs scores (and all of these computations have $O(n \log n)$ complexity). On the other hand, the original estimator based on the trimmed Mann-Whitney U statistic in Eq. (14), has computational complexity of order $O(n_y n_x)$ as it relies on a double summation running across all (*i*, *j*) pairs for $i = 1, \ldots , n_x$ and $j = 1, \ldots , n_y$. Note that this $O(n_y n_x)$ complexity is most expensive for balanced populations where $n_x = n_y$ as corroborated in our experiments (Fig. 6a), where the results from the experiments based on $n_x = n_y$ took more time to compute.

We also compare the proposed estimator ($\widehat{tpAUC}_p$, in Eq. 24) against the alternative estimator ($\widehat{tpAUC}_a$, in Eq. 26), which is simply a direct implementation of the mathematical definition of tpAUC in Eq. 12 (originally proposed by [16]) using the pROC package. Our comparisons (Figs. 7b and 8b) show that while the alternative estimator can be slightly faster than the proposed one, the small differences are unimportant in practice. (Note that the alternative estimator also has a $O(n \log n)$ computational complexity.)

The R code used to implement the proposed estimator and reproduce all the experiments presented in this paper is available in the github repository, https://github.com/echaibub/pROC_based_tpAUC. Additionally, the github repository https://github.com/Sage-Bionetworks/tp_AUC provides scripts to run the R code in the Python environment (using the rpy2 Python library^1^) and makes the proposed estimator available to the Python users community as well.

## Conclusions

In summary, as clearly demonstrated by our synthetic and real data illustrations, our proposed tpAUC estimator represents a computationally efficient alternative to the original estimator based on trimmed Mann-Whitney U statistic for moderate to large datasets, which also tends to be less biased in small datasets. But most importantly, the proposed estimator makes the calculation of bootstrap-based confidence intervals feasible, and opens the doors for the comparison of diagnostic tests/machine learning classifiers in large datasets where the serial computation of the original estimator is impractical.

## Data Availability

The diabetic retinopathy and sepsis survival datasets used in this paper are available in the UCI Machine Learning repository and can be downloaded, respectively, from https://archive.ics.uci.edu/ml/datasets/Diabetic+Retinopathy+Debrecen+Data+Set and https://archive.ics.uci.edu/ml/datasets/Sepsis+survival+minimal+clinical+records. The code for reproducing all the results in this paper is available in https://github.com/echaibub/pROC_based_tpAUC. Scripts for running the R code in the Python environment are provided in https://github.com/Sage-Bionetworks/tp_AUC.

## Abbreviations

AUC: Area under the curve
$pAUC_{se}$: Partial AUC focusing on sensitivity
$pAUC_{sp}$: Partial AUC focusing on specificity
$pAUC_{fpr}$: Partial AUC focusing on false positive rate
ROC: Receiver operating characteristic curve
*tpAUC*: Two-way partial AUC
$b_{se}$: Sensitivity bound or threshold
$b_{sp}$: Specificity bound or threshold
*ML*: Machine learning
*TPR*: True positive rate
*FPR*: False positive rate
*SENS*: Sensitivity
*SPEC*: Specificity

## Footnotes

1 https://rpy2.github.io/doc/latest/html/index.html

## References

[1] Pepe MS (2003). The statistical evaluation of medical tests for classification and prediction.

[2] Jordan MI, Mitchell TM (2015). Machine learning: Trends, perspectives, and prospects. Science..

[3] Doi K. Computer-aided diagnosis in medical imaging: historical review, current status and future potential. Comput Med Imaging Graph. 2007;31:198-201. 10.1016/j.compmedimag.2007.02.002 PMC1955762 17349778

[4] Mookiah MRK, Acharya UR, Chua CK, Lim CM, Ng EYK, Laude A (2013). Computer-aided diagnosis of diabetic retinopathy: a review. Comput Biol Med..

[5] Goncalves VM, Delamaro ME, Nunes FLS (2014). A systematic review on the evaluation and characteristics of computer-aided diagnosis systems. Braz J Biomed Eng..

[6] Fujita H (2020). AI-based computer-aided diagnosis (AI-CAD): the latest review to read first. Radiol Phys Technol..

[7] Chan HP, Hadjiiski LM, Samala RK (2020). Computer-aided diagnosis in the era of deep learning. Med Phys..

[8] Fawcett T (2006). An introduction to ROC analysis. Pattern Recogn Lett..

[9] Linnet K (1987). Comparison of quantitative diagnostic tests type I error, power, and sample size. Stat Med..

[10] Swets JA (1988). Measuring the accuracy of diagnostic systems. Science.

[11] Hanley JA (1989). Receiver operating characteristic (ROC) methodology: the state of the art. Crit Rev Diagn Imaging..

[12] Begg CB (1991). Advances in statistical methodology for diagnostic medicine in the 1980’s. Stat Med..

[13] Zhou XH, Obuchowski NA, McClish DK (2011). Statistical methods in diagnostic medicine.

[14] World Health Organization. High priority target product profiles for new tuberculosis diagnostics: report of a consensus meeting, 28-29 April 2014, Geneva, Switzerland. World Health Organization; 2014. https://apps.who.int/iris/handle/10665/135617.

[15] World Health Organization. Antigen-detection in the diagnosis of SARS-CoV-2 infection: Interim guidance, 6 October 2021. World Health Organization; 2021. https://iris.who.int/handle/10665/345948.

[16] Yang H, Lu K, Lyu X, Hu F (2019). Two-way partial AUC and its properties. Stat Methods Med Res..

[17] Dodd LE, Pepe MS (2003). Partial AUC estimation and regression. Biometrics..

[18] McClish DK (1989). Analysing a portion of the ROC curve. Med Dec Making..

[19] Zhang DD, Zhou XH, Freeman DH (2002). A non-parametric method for the comparison of partial areas under ROC curves and its application to large health care data sets. Stat Med..

[20] Wang X, Ma J, George S (2012). Estimation of AUC or partial AUC under test-result-dependent sampling. Stat Biopharm Res..

[21] Ma H, Bandos AI, Rockette HE (2013). On use of partial area under the ROC curve for evaluation of diagnostic performance. Stat Med.

[22] Mann HB, Whitney DR. On a test of whether one of two random variables is stochastically larger than the other. Ann Math Stat. 1947;18:50–60.

[23] R Core Team. R: A language and environment for statistical computing. Vienna: R Foundation for Statistical Computing; 2021. https://www.R-project.org/.

[24] Efron B, Tibshirani R. An Introduction to the Bootstrap. Chapman & Hall/CRC Monographs on Statistics and Applied Probability. Philadelphia: Chapman & Hall/CRC; 1994.

[25] Robin X, Turck N, Hainard A, Tiberti N, Lisacek F, Sanchez JC (2011). pROC: an open-source package for R and S+ to analyze and compare ROC curves. BMC Bioinformatics..

[26] Bamber D (1975). The area above the ordinal dominance graph and the area below the receiver operating characteristic graph. J Math Psychol..

[27] Thompson ML, Zucchini W (1989). On the statistical analysis of ROC curves. Stat Med..

[28] Jiang Y, Metz C, Nishikawa R (1996). A receiver operating characteristic partial area index for highly sensitive diagnostic tests. Radiology..

[29] Antal B, Hajdu A (2014). An ensemble-based system for automatic screening of diabetic retinopathy. Knowl-Based Syst..

[30] Chicco D, Jurman G (2020). Survival prediction of patients with sepsis from age, sex, and septic episode number alone. Sci Rep..

[31] Dua D, Graff C. UCI Machine Learning Repository [http://archive.ics.uci.edu/ml]. Irvine: University of California, School of Information and Computer Science; 2019.

[32] Decenciere E, Zhang X, Cazuguel G, Lay B, Cochener B, Trone C (2014). Feedback on a publicly distributed image database: the Messidor database. Image Anal Stereol..

[33] Cox DR (1958). The regression analysis of binary sequences. J R Stat Soc Ser B Methodol..

[34] Breiman L (2001). Random forests. Mach Learn..

[35] Wright MN, Ziegler A (2017). ranger: A Fast Implementation of Random Forests for High Dimensional Data in C++ and R. J Stat Softw. 2017;77:1-17. 10.18637/jss.v077.i01.

[36] Hanley JA, McNeil BJ (1982). The meaning and use of the area under a receiver operating characteristic (ROC) curve. Radiology..

[37] Hajian-Tilak KO, Hanley JA, Joseph L, Collet JP (1997). A comparison of parametric and non-parametric approaches to ROC analysis of quantitative diagnostic tests. Med Decis Making..

